# Neural Network-Assisted DPD of Wideband PA Nonlinearity for Sub-Nyquist Sampling Systems

**DOI:** 10.3390/s25041106

**Published:** 2025-02-12

**Authors:** Mengqiu Liu, Xining Yang, Jian Gao, Sen Cao, Guisheng Liao, Gaopan Hou, Dawei Gao

**Affiliations:** 1 Hangzhou Institute of Technology, Xidian University, Hangzhou 311200, China; lmengqiu@stu.xidian.edu.cn (M.L.); liaogs@xidian.edu.cn (G.L.); hougaopan@xidian.edu.cn (G.H.); 2 29th Research Institute of China Electronics Technology Group Corporation, Chengdu 610036, China; yangxining@cetc.com.cn (X.Y.); swieegaoj@sina.com (J.G.); caosen@cetc.com.cn (S.C.)

**Keywords:** digital predistortion (DPD), undersampling, memory polynomial, attention mechanism model

## Abstract

The design of conventional digital predistortion (DPD) requires an analogue-to-digital converter (ADC) with a sampling frequency that is multiple times the signal bandwidth, which is extremely challenging for sub-Nyquist sampling systems with undersampled signals. To address this, this paper proposes a neural network (NN)-assisted wideband power amplifier (PA) DPD method for sub-Nyquist sampling systems, wherein a dual-stage architecture is designed to handle the ambiguity caused by subsampled communications signals. In the first stage, the time-delayed polynomial reconstruction method is employed to estimate the wideband DPD nonlinearity coarsely with the undersampled signals with limited pilots. In the second stage, an NN-based DPD method is proposed for the virtual training of the DPD, which learns the up-sampled DPD behavior by taking advantage of the pre-estimated DPD model and the input data signals, which reduces the length of the training sequence significantly and refines the DPD behavior efficiently. Simulation results demonstrate the efficacy of the proposed method in tackling the wideband PA nonlinearity and its ability to outperform the conventional method in terms of power spectrum, error vector magnitude, and bit error rate.

## 1. Introduction

With the rapid development of sixth-generation (6G) wireless communication, the spectrum width is increasing rapidly. This trend necessitates the application of ultra-wideband communication and spectrum sensing technologies. However, the elevated frequency band makes the nonlinearity of power amplifiers (PAs) severer than ever, where the nonlinearity becomes frequency-dependent, and memory effects need to be taken into account [1]. The nonlinear distortion of the wideband PA leads to poor signal detection performance, which needs to be handled appropriately [2]. Digital predistortion (DPD) techniques are proposed to mitigate the nonlinear distortion effectively [3,4], but the design of DPD usually requires three to five times the bandwidth of the input signal. This presents a significant challenge for broadband communications. In particular, when sub-Nyquist-sampled systems are employed for low-cost and efficient implementation using severely undersampled, low-speed ADCs (analogue-to-digital converters), conventional digital predistortion (DPD) methods cannot be applied directly [5]. Therefore, this study investigates the DPD method in the context of sub-Nyquist sampling of communication systems to achieve effective nonlinear correction of amplifiers, even when ADCs are undersampled.

Spectral extrapolation, a commonly used undersampling predistortion method proposed in [6], models the power amplifier (PA) under band-limited sampled data to recover or approximate the original output signal that should be sampled based on the estimated PA model. However, this approach has constraints that necessitate the inclusion of a band-limited filter in the feedback channel, which can be problematic in practical situations due to the performance of the band-limited filter and the challenges in its design. In [7], an undersampling recovery digital predistortion technique (USR-DPD) is proposed, which iteratively recovers the full-band output signal from the undersampled output signal, and then extracts the DPD parameter information using a memory model. However, this method requires the addition of extra DACs to the system, thereby increasing the system’s complexity. Moreover, the method requires multiple internal and external iterations to obtain the final estimated DPD signal, which increases the computational cost. Ref. [8] proposed a forward behavior modeling approach that utilizes low-rate aliased PA output signals to estimate the DPD model coefficients. In this approach, the aliased low-rate PA output signals are employed to estimate the model parameters across the full frequency band of the PA. In [5], an undersampling digital predistortion method based on a multi-tone mixing feedback technique (MTM-DPD) is proposed for the processing of multi-tone mixing under undersampling conditions. In [9], a novel undersampled feedback signal iterative learning control (US-ILC-DPD) technique is proposed. This technique still exhibits good PA nonlinearity compensation even at low sampling rates. In [10], a random demodulation-based reduced sampling rate (RDRS) method is proposed to recover the full-band power amplifier (PA) output signal. This method uses only the I-way component of the band-limited feedback signal to reconstruct the full-band signal output. Although the method has a simple structure, it introduces a time alignment problem due to the additional random sequence generator and analog multiplier in the feedback path. This issue still needs to be addressed to ensure accurate signal reconstruction. In [11], a novel predistortion technique is proposed, which consists of a memoryless amplitude-to-amplitude (AM/AM) gain function that can be implemented in the analog domain and a nonlinear model with memory effects in the digital domain, reducing the sampling rate requirements of the forward and feedback paths. However, most of these methods have significant limitations. They are particularly vulnerable to noise and often fail to deliver satisfactory performance under the conditions of low signal-to-noise ratios (SNRs).

Artificial neural networks (NNs) have been rapidly developed in recent years and can be used for PA and DPD modeling as they can exhibit strong nonlinear feature representation without pre-conditioning [12,13]. Ref. [14] proposed a method for combining recurrent neural networks with PA modeling and DPD. Ref. [15] proposed the application of long short-term memory (LSTM) networks in the fields of amplifier modeling and digital predistortion. However, these models do not account for the downsampling environment. In [16], a digital predistorter, modeled by an augmented real-valued time delay neural network (ARVTDNN), was proposed to realize the correction of PA nonlinearity in broadband transmitters.The structure of this network is simple. However, its application scenarios are limited, and its performance decreases in some specific scenarios. In [17], a nonlinear correction method for power amplifiers is proposed, which combines a global optimization search algorithm with adaptive linearization to adaptively find the best parameters for nonlinear correction. In [18], a deep neural network (DNN) model was proposed for low-feedback estimation of DPD. However, this approach first requires the recovery of the model parameters of the PA before solving for the inverse model coefficients. This additional step increases the overall system complexity. Attentional mechanisms are inspired by the human visual system and are widely used in neural network models for their ability to improve model performance and model flexibility and generalization [19]. However, the method does not account for sub-Nyquist sampling scenarios. Moreover, the sparse characteristics of the undersampled data stream can negatively impact the training process of neural networks (NNs). This issue is demonstrated in the simulation results and needs to be carefully addressed.

This paper proposes an NN-assisted wideband PA DPD method for sub-Nyquist sampling communication systems, where a dual-stage architecture is designed to handle the ambiguity caused by the down-sampled signals. In the first stage, a time-delayed memory polynomial reconstruction (TDMPR)-based method is implemented with the undersampled signals for the preliminary estimation of DPD parameters, which demands a limited number of pilot signals. Then, an attention-based NN is proposed for the refinement of DPD, where virtual training is performed with transmitted data signals, reducing the training overhead significantly. It is demonstrated through simulations that the proposed method can mitigate the wideband PA nonlinearity efficiently for sub-Nyquist sampling systems while providing outstanding performance gains compared to the existing downsampling DPD methods.

## 2. Signal Model

**Figure 1:**
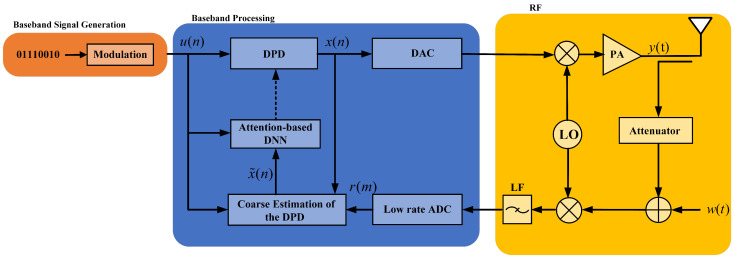
A system block diagram of the sub-Nyquist sampling system.

We consider a single-user uplink communications system. Considering the cost of mobile devices, we assume that the user has a single antenna where low-cost PA is employed, resulting in nonlinear distortion with memory effects. The feedback channel is equipped with a low-rate ADC. The system block diagram is shown in Figure 1.

The modulated signal u(n) is first transmitted through a DPD module to generate $x\left(n\right)=f_{\mathrm{DPD}}\left(u\left(n\right)\right)$, where $f_{\mathrm{DPD}}\left(\cdot \right)$ denotes the transfer function of DPD, and the learning of the DPD is the focus of this work. The x(n) is first upconverted, then passed through the DAC, and, finally, fed into the PA. To represent the wideband PA’s nonlinear behavior, the memory polynomial (MP) model is employed [20]. The MP model is represented as a baseband signal with a discrete form, i.e.,

(1) $$y\left(n\right)=\sum_{\begin{array}{c} k=1 \end{array}}^{K}\sum_{q=0}^{Q}h_{kq}x\left(n-q\right){\left|x\left(n-q\right)\right|}^{k-1},$$

where *Q* is the maximum memory benefit depth, *K* is the maximum nonlinear order, and *k* is only odd, while $h_{kq}$ is the coefficient of the polynomial, and n=0,1,…,N−1. The reason for implementing only odd orders is that the main factor of nonlinear distortion is the spectral aliasing and inter-modulation distortion produced by odd orders, so the effect of even orders can be ignored. (1) can be written in matrix form as follows:

(2) y=Xh,

where

(3) $$y={\left[y(0),y(1),...,y(N-1)\right]}^{T}$$

(4) $$h={\left[h_{10},\ldots ,h_{1Q},h_{30},\ldots ,h_{3Q},\ldots ,h_{K0},\ldots ,h_{KQ}\right]}^{T}.$$

Define $\varphi_{kq}\left(n\right)=x\left(n-q\right){\left|x\left(n-q\right)\right|}^{k-1}$, and the matrix X is represented as

(5) $$X\;=\;\left[\begin{array}{cccc} \varphi_{10}\left(0\right) & \varphi_{11}\left(0\right) & \cdots & \varphi_{KQ}\left(0\right)\\ \varphi_{10}\left(1\right) & \varphi_{11}\left(1\right) & \cdots & \varphi_{KQ}\left(1\right)\\ \vdots & \vdots & \; & \vdots \\ \varphi_{10}\left(N-1\right) & \varphi_{11}\left(N-1\right) & \cdots & \varphi_{KQ}\left(N-1\right) \end{array}\right].$$

The PA output signal y(n) is transmitted through the wireless channel to obtain r(n). Therefore, the feedback signal r(n) at the BS can be expressed as

(6) $$r\left(n\right)=y\left(n\right)+\omega \left(n\right),$$

where $\omega \left(n\right)$ is the noise signal, which is introduced in the feedback channel and is assumed to be Gaussian for simplicity. Due to the nonlinearity of the wideband PA, the spectrum of the feedback signal r(n) in the receiver is broadened, so an ADC with high sampling frequency is required to achieve unambiguous sampling. However, a sub-Nyquist sampling system, an ADC with a sampling rate that is much smaller than the Nyquist sampling frequency, is used to sample the feedback signal r(n). The rate of the undersampled ADC is 1/D (*D* is an integer, and D>0) of the Nyquist sampling frequency. r(n) is processed by the low-rate ADC, resulting in r(Dn). Hence, our aim is to restore the transmitted signal u(n) from the undersampled feedback signals r(Dn) by designing a novel DPD architecture with limited pilots, as detailed in Section 3.

## 3. Proposed NN-Assisted Dual-Stage DPD for Sub-Nyquist Sampling Systems

### 3.1. Stage 1: Time-Delayed Memory Polynomial Reconstruction-Based Coarse Estimation of the Digital Predistorter

**Figure 2:**
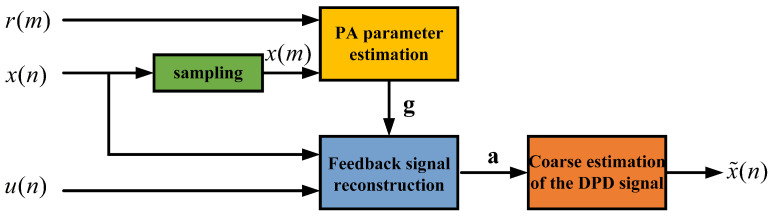
The structure of the proposed attention-based NN for virtual training.

Directly using downsampled feedback signals for training a neural network model will prevent the model from accurately learning the behavior of the Digital Predistortion (DPD) system, leading to poor performance. Therefore, a preliminary estimation of the DPD signals is necessary before model training [8]. As depicted in Figure 1, after the feedback signal passes through a low-rate ADC, it must be combined with the high-rate complex baseband signal and fed into the DPD signal coarse estimation module, the structure of which is shown in Figure 2. The downsampled feedback signal is denoted as r(m), and r(m)=r(Dn). The complex baseband signal x(n) is first sampled to obtain x(m), where x(m)=x(Dn). Subsequently, the PA forward model is constructed using both r(m) and x(m). The expression for the PA forward model is

(7) $$r\left(m\right)=\sum_{\begin{array}{c} k=1 \end{array}}^{K}\sum_{q=0}^{Q}g_{kq}x\left(m-q\right){\left|x\left(m-q\right)\right|}^{k-1},$$

where *K* and *Q* are the same as in (1). Therefore, this PA model can also be expressed in a matrix form, like (2):

(8) $$r=X_{D}g,$$

where

(9) $$r={\left[r(0),r(1),\ldots ,r(M-1)\right]}^{T}$$

(10) $$g={\left[g_{10},\ldots ,g_{1Q},g_{30},\ldots ,g_{3Q},\ldots ,g_{K0},\ldots ,g_{KQ}\right]}^{T}.$$

The matrix $X_{D}$ is represented as

(11) $$X_{D}\;=\;\left[\begin{array}{cccc} \varphi_{10}\left(0\right) & \varphi_{11}\left(0\right) & \cdots & \varphi_{KQ}\left(0\right)\\ \varphi_{10}\left(1\right) & \varphi_{11}\left(1\right) & \cdots & \varphi_{KQ}\left(1\right)\\ \vdots & \vdots & \; & \vdots \\ \varphi_{10}\left(M-1\right) & \varphi_{11}\left(M-1\right) & \cdots & \varphi_{KQ}\left(M-1\right) \end{array}\right].$$

Using least square (LS) solutions, the coefficients g can be obtained as

(12) $$g={\left(X_{D}^{H}X_{D}\right)}^{-1}X_{D}^{H}r.$$

g is a parameter of the new PA model, which is constructed using the downsampling signal. With this new power amplifier model, a reconstructed feedback signal $r_{rec}\left(n\right)$ based on a storage polynomial is obtained according to (2), where the parameter h is replaced by g. A preliminary DPD model is built using the reconstructed feedback signal $r_{rec}\left(n\right)$ and the high-rate baseband complex signal x(n). Like (2) and (8), it can be expressed in matrix form:

(13) $$x=R_{rec}a,$$

where x is an *N*-dimensional column vector, a is a column vector with (K+1)/2∗(Q+1) elements, and $R_{rec}$ is a N×(K+1)/2∗(Q+1) matrix, which is derived in the same way as in Section 2. Using least square (LS) solutions, the coefficients a can be obtained as

(14) $$a={\left(R_{rec}^{H}R_{rec}\right)}^{-1}R_{rec}^{H}x.$$

Based on the determined a, the coarse estimation of the DPD behavior can be determined, and it can be used for the prediction of the DPD output whenever data are incorporated.

### 3.2. Stage 2: An Attention-Based NN for the Virtual Training of DPD

Based on the coarse estimate of DPD from Stage 1, we propose an attention-based NN to refine the DPD, where virtual training is implemented with the input data and the trained MP model. We assume we have the new data $\tilde{u}\left(n\right)$ coming in. Before refining the DPD model, the DPD output can be predicted based on a, which is calculated in the first stage, as expressed below:

(15) $$\tilde{x}\left(n\right)=\sum_{\begin{array}{c} k=1 \end{array}}^{K}\sum_{q=0}^{Q}a_{kq}u\left(n-q\right){\left|u\left(n-q\right)\right|}^{k-1}.$$

With the data pairs ${\left\{\left(\tilde{u}\left(n\right),\tilde{x}\left(n\right)\right)\right\}}_{n=0}^{N_{0}-1}$, we proposed an attention-based NN to refine the DPD behavior and used the pairs for the virtual training of the NN.

**Figure 3:**
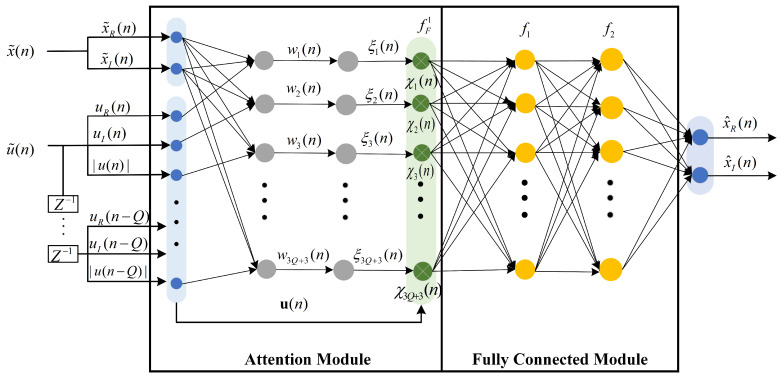
The structure of the attention-based NN proposed for virtual training.

Figure 3 illustrates the structure of the proposed NN. The operation of each layer is given in the following subsections.

#### 3.2.1. Inputs and Outputs of the Proposed NN

The model’s inputs encompass the real, imaginary, and norm of the current and historical input signals of the DPD, as well as the real and imaginary output signals of the DPD estimated from Stage 1. Consequently, the inputs can be represented as

(16) $$\tilde{u}\left(n\right)=[u_{R}\left(n\right),u_{I}{\left(n\right),|u\left(n\right)|,\cdots ,|u\left(n-Q\right)|]}^{T},$$

where $u_{R}\left(n\right)$ represents the real part of u(n), $u_{I}\left(n\right)$ represents the imaginary part of u(n), |u(n)| is denoted as the norm of u(n), and *Q* signifies the depth of memory. The NN’s output is the estimated DPD signal with the real and imaginary parts separately, i.e.,

(17) $$\tilde{x}\left(n\right)={\left[{\tilde{x}}_{R}\left(n\right),{\tilde{x}}_{I}\left(n\right)\right]}^{T}.$$

#### 3.2.2. Attention Module

The front part of the model comprises an attention mechanism featuring two layers. The first one is a nonlinear layer with the activation function tanh, where the size is equal to the inputs $\tilde{u}\left(n\right)$. Hence, the *i*-th output from the first layer of the attention module is represented as

(18) $$w_{i}\left(n\right)=tanh\left(\alpha_{i}^{T}\tilde{u}\left(n\right)+\beta_{i}^{T}\tilde{x}\left(n\right)+b_{i}\right),$$

where $w_{i}\left(n\right)$ denotes the correlation between the *i*-th input and the pre-estimated DPD output, and u(n) denotes the data input at the time instant *n*. $\left\{\begin{array}{c} \alpha_{i},\beta_{i},b_{i} \end{array}\right\}$ are the parameters in the attention module. The second layer is a weight calculation layer, which is utilized to calculate the weight of each input neuron and reduce the importance of the redundant estimated signals. The output from the second layer of the attention module is denoted as

(19) $$\xi_{i}\left(n\right)=\frac{exp(w_{i}\left(n\right))}{\sum_{i=1}^{3Q+3}exp\left(w_{i}\left(n\right)\right)},\left(i=1,2,\cdots ,3Q+3\right).$$

Multiplying the calculated weights by the input $U_{n}$ yields the weighted signal, as shown below:

(20) $$\chi_{i}\left(n\right)=\xi_{i}\left(n\right){\tilde{u}}_{i}\left(n\right).$$

#### 3.2.3. Fully Connected Module

The weighted signal output from the attention module is then input to a module with a fully connected layer for regression. The module contains three hidden layers. All hidden layers use the tanh activation functions. The output of the fully connected module can be simply expressed as

(21) $$\hat{x}\left(n\right)=tanh\left(C_{2}tanh\left(C_{1}\chi \left(n\right)+b_{1}\right)+b_{2}\right),$$

where $\chi \left(n\right)=[\chi_{1}\left(n\right),\chi_{2}\left(n\right),\ldots ,\chi_{3Q+3}\left(n\right)]$, and $C_{1},C_{2},b_{1},b_{2}$ denotes the parameters of the hidden layer, where $C_{1}$ can be denoted as

(22) $$C_{1}\;=\;\left[\begin{array}{cccc} c_{1,11} & c_{1,12} & \cdots & c_{1,1(3Q+3)}\\ c_{1,21} & c_{1,22} & \cdots & c_{1,2(3Q+3)}\\ \vdots & \vdots & \; & \vdots \\ c_{1,L_{1}1} & c_{1,L_{1}2} & \cdots & c_{1,L_{1}\left(3Q+3\right)} \end{array}\right].$$

and $b_{1}$ can be denoted as

(23) $$b_{1}={\left[b_{1,1},b_{1,2},b_{1,3},\cdots ,b_{1,L_{1}}\right]}^{T}.$$

Similarly, $C_{2}$ is expressed as

(24) $$C_{2}\;=\;\left[\begin{array}{cccc} c_{2,11} & c_{2,12} & \cdots & c_{2,1(3Q+3)}\\ c_{2,21} & c_{2,22} & \cdots & c_{2,2(3Q+3)}\\ \vdots & \vdots & \; & \vdots \\ c_{2,L_{2}1} & c_{2,L_{2}2} & \cdots & c_{2,L_{2}\left(3Q+3\right)} \end{array}\right],$$

and $b_{2}$ is expressed as

(25) $$b_{2}={\left[b_{2,1},b_{2,2},b_{2,3},\cdots ,b_{2,L_{2}}\right]}^{T},$$

where $L_{1}$ and $L_{2}$ are the number of nodes in the first hidden layer and the second hidden layer, respectively. tanh() is the activation function, which has good approximation ability for DPDs [21], and it can be expressed as

(26) $$tanh\left(x\right)=\frac{1-exp(-2x)}{1+exp(-2x)}.$$

The proposed attention-based NN is trained using the Adam optimization algorithm and the loss of mean square error (MSE) to tune the parameters of the model, where the loss function is

(27) $$Loss=\frac{1}{2}\frac{1}{N_{0}}\sum_{n=0}^{N_{o}-1}\sum_{i=1}^{2}{\left({\hat{x}}_{i}\left(n\right)-{\tilde{x}}_{i}\left(n\right)\right)}^{2}.$$

The learning rate is set to 0.085. The specific steps of the proposed method are outlined in Algorithm 1.
**Algorithm** **1** The Proposed Dual-Stage DPD Method for sub-Nyquist Sampling Systems**Input**     1. Pilot signals: PA input signal x, Downsampled PA input signal $x_{D}$, feedback signal r.     2. Data: $\left\{\tilde{u}\left(n\right)\right\}$.**Stage** **1:**   1. PA nonlinear model reconstruction using sub-nyquist Nyquist sampling data and calculate the polynomial coefficients:                                 $g={\left(X_{D}^{H}X_{D}\right)}^{-1}X_{D}^{H}r$.   2. Reconstruction of the feedback signal from down-mining using the estimated PA model parameters:                                 $r_{\mathrm{rec}}=Xg$.   3. The pre-estimated DPD parameters are obtained by building a PA inverse model based on the reconstructed feedback signal:                                 $a={\left(R_{rec}^{H}R_{rec}\right)}^{-1}R_{rec}^{H}x.$**Stage** **2:**   Virtual training:     1. Generate expected DPD outputs using data:            $\tilde{x}\left(n\right)=\tilde{u}\left(n\right)a,n=0,1,\ldots ,N_{0}-1$.     2. Virtual training of the DPD:       (1) Formulate the inputs and outputs of the proposed NN using (16) and (17);       (2) Calculate the output of the attention module as described in (20);       (3) Determine the output of the proposed NN based on (21);       (4) Update the hyper parameters according to the loss function in (27).**Predicting**   Using the train NN to predict $\hat{x}\left(n\right)$.

## 4. Simulation Results

In this section, we conduct simulations to demonstrate the effectiveness of the proposed NN-assisted wideband PA DPD method. We consider a single-user uplink communications system. The 16-QAM modulated signal is generated. The baseband waveforms are generated by up-sampling five times and then passing through a raised cosine filter with a roll-off coefficient of 0.25. For the wideband PA modeling, the MP model is simulated with nonlinear order K=5, and its memory depth is Q=2. According to [22], the coefficients are $\left[h_{10},h_{30},h_{50},h_{11},h_{31},h_{51},h_{12},h_{32},h_{52}\right]=\left[1.0513+0.0904j,-0.0542-0.2900j,-0.9657-0.7028j,-0.0680-0.0023j,0.2234+0.2317j,-0.2451-0.3735j,0.0289-0.0054j,-0.0621-0.0932j,0.1229+0.1508j\right]$. For the MP-based DPD estimation in Stage 1, the order and the memory remain the same. At the feedback channel, the feedback signal is undersampled by a factor of D=4, which means that the ADC sampling rate only needs to be 1/4 of the Nyquist sampling frequency. For the proposed NN, the number of nodes in the hidden layers is [10,5]. Regarding the number of training sets, 1000 and 100,000 sets of data are used for validation. The number of Monte Carol runs is 500. For the evaluation metrics, the EVM is defined as

(28) $$\mathrm{EVM}=\sqrt{\frac{\frac{1}{N}\sum_{n=0}^{N-1}{\left|\hat{x}\left(n\right)-x_{t}\left(n\right)\right|}^{2}}{\frac{1}{N}\sum_{n=0}^{N-1}{\left|x_{t}\left(n\right)\right|}^{2}}},$$

where *N* denotes the number of symbols measured by the EVM, and $\hat{x}\left(n\right)$ denotes the *n*-th normalized feedback symbol, while $x_{t}\left(n\right)$ denotes the ideal value of the *n*-th symbol. The SNR is defined as

(29) $$\mathrm{SNR}\left(\mathrm{dB}\right)=10lg\left(\frac{P_{\mathrm{sig}\;}}{P_{\mathrm{noi}\;}}\right),$$

where $P_{\mathrm{sig}}$ indicates signal power, and $P_{\mathrm{noi}}$ indicates noise power. Furthermore, the adjacent channel power ratio (ACPR) can be used to describe the power spectrum leakage into neighboring channels. It is expressed as the ratio of the leakage power in the neighboring channel to the signal power in the reference channel. A larger ACPR value indicates more severe leakage and, consequently, more serious nonlinear distortion. Since leakage can occur in both the upper and lower neighboring channels, the ACPR can be calculated separately for each. The upper neighborhood ACPR is expressed as

(30) $$ACPR_{u}\left(dB\right)=\frac{P_{ACH}}{P_{REFC}}=10lg\frac{\int_{ACH}P\left(f\right)df}{\int_{REFC}P\left(f\right)df},$$

where $P_{ACH}$ is the power of the signal in the upper neighboring channel, and $P_{REFC}$ is the signal power in the reference channel. Similarly, the lower neighborhood ACPR can be expressed as

(31) $$ACPR_{l}\left(dB\right)=\frac{P_{ACL}}{P_{REFC}}=10lg\frac{\int_{ACL}P\left(f\right)df}{\int_{REFC}P\left(f\right)df},$$

where $P_{ACL}$ is the power of the lower neighboring channel signal.

In order to validate the effectiveness of the proposed method, the proposed method is compared with the augmented real-valued time delay neural network (ARVTDNN) method proposed in [16]. The ARVTDNN is an augmented real-valued time delay neural network. The ARVTDNN consists of an input layer, a hidden layer, and an output layer. The inputs to the network are the Cartesian components (I/Q) of the input signals and the envelope correlation. The network has only one hidden layer with 17 nodes, and the output of the network consists of the I and Q paths of the estimated signal. The simulation setups of the ARVTDNN in the simulations are the same as in [16]. We also simulated the method presented in [8], for which we employed time-delayed memory polynomial reconstruction for sub-nyquist-sampled DPD, denoted as TDMPR.

**Figure 4:**
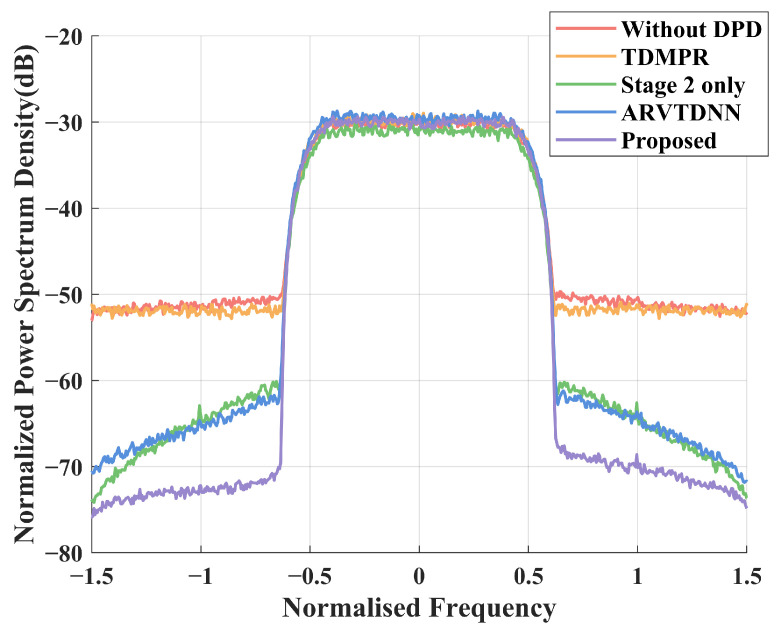
PSD performance of different methods at SNR=15dB.

Figure 4 depicts the power spectrum density (PSD) of various DPD methods, including the proposed NN-based method, the ARVTDNN, TDMPR, a method based on the direct application of the DNN proposed in Stage 2, and a method without a DPD function, where the SNR is set as 15dB.

It can be clearly seen that without DPD, the out-of-band spectrum growth cannot be suppressed at all, which is significantly detrimental to the communication performance. The PSD with the TDMPR-based DPD achieves a marginal performance gain compared to the one without DPD, which is due to the numerical instability of the MP, even with the aid of virtual data sequences. For the direct DNN-based method, the lack of processing of the downsampled feedback signals results in the network being unable to correctly learn the nonlinear behavior of the DPD, leading to a poor nonlinear correction capability. In contrast, our proposed NN-assisted DPD method provides significant performance gains compared to the other methods. And the spectrum leakage is significantly improved with the proposed method compared to the method in [16].

Table 1 compares the ACPR and EVM of DPD for various methods at SNR=15 dB. It can be seen that the proposed method has outstanding performance and reduces the ACPR from −20 dB to −42 dB. The ARVTDNN reduced the ACPR by 16 dB, which is effective but still limited compared to the proposed method. The performance of the DNN proposed in the method based on Stage 2 is close to that of the ARVTDNN, while the lack of coarse estimation leads to performance loss, which speaks to the efficiency of the proposed two-stage methods. In addition, TDMPR is poorly corrected at low SNRs, with ACPR being reduced by only 2 dB. In conclusion, it is evident that the proposed method has excellent corrective capabilities compared to the state-of-the-art methods.

**Figure 5:**
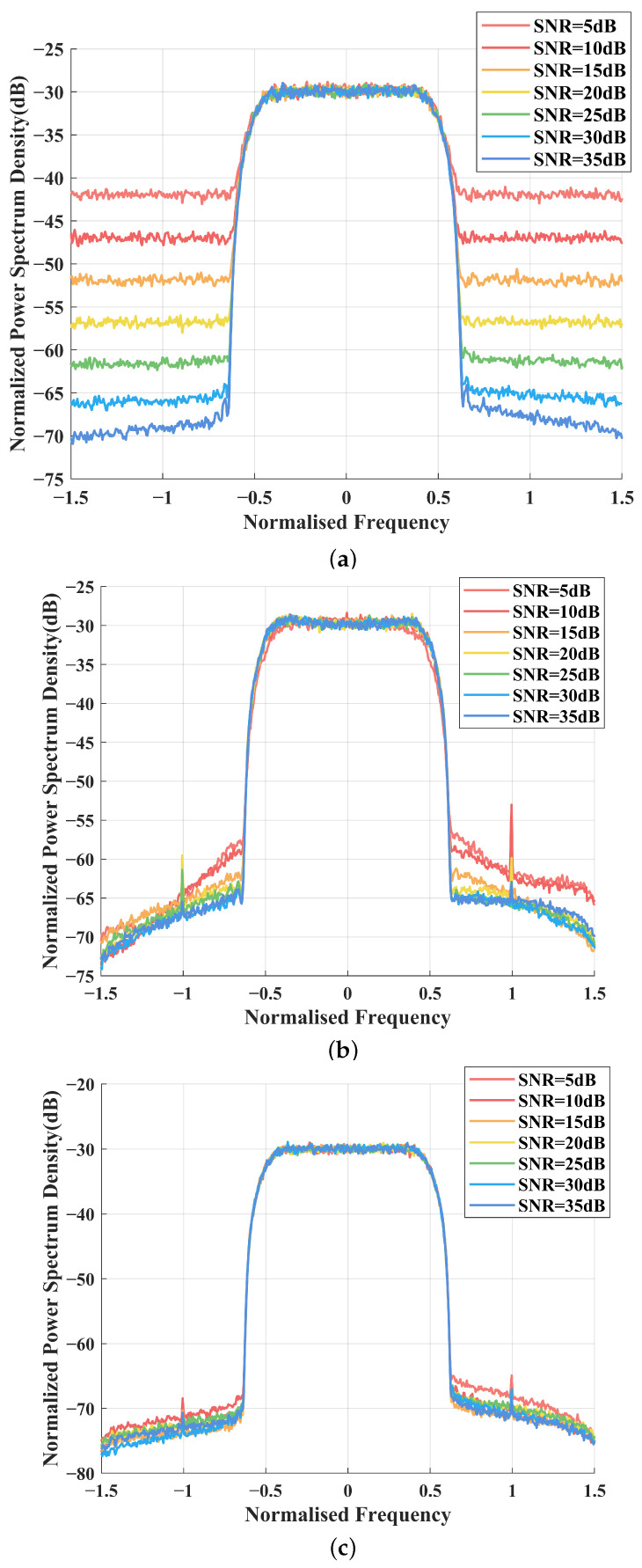
PSD performance of various DPD methods versus different SNRs: (**a**) PSD of PA output with TDMPR, (**b**) PSD of PA output with ARVTDNN, and (**c**) PSD of PA output with proposed NN-assisted DPD.

To observe the detailed variations of these DPD methods under different SNRs, we simulated the proposed DPD method, TDMPR, and the method without DPD, and the results are shown in Figure 5. The SNR is from 5 dB to 35 dB. It can be seen that the proposed NN-assisted method has spectral bands without leakage along various SNR points, which demonstrates the robustness of the proposed method even under low SNRs. Although ARVTDNN also has good noise immunity, this method does not perform as well as the proposed method. In contrast, TDMPR provides unstable PSD performance and marginal performance gain. This suggests that relying solely on TDMPR for estimating the DPD signal is insufficient for mitigating the nonlinear effects induced by the PA, particularly when the SNR is low and the signal is undersampled.

**Figure 6:**
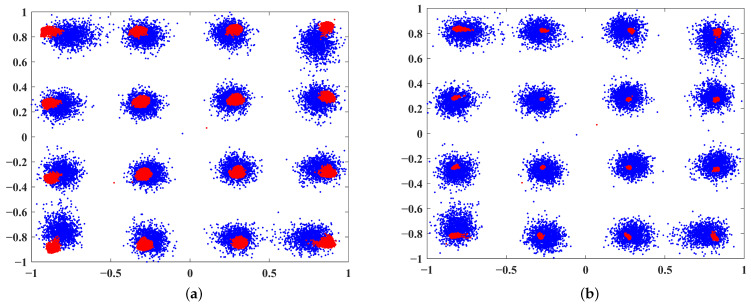
Constellation diagrams at SNR=15dB: (**a**) ARVTDNN and (**b**) the proposed method.

The effects of DPD can be more intuitively observed in Figure 6. The blue constellation diagram represents the signal before correction, showing significant distortion after passing through the PA. In contrast, the red constellation diagram represents the signal after correction. It is evident that the proposed method’s distortion correction is significantly better than that of ARVTDNN, indicating superior PA correction performance.

**Figure 7:**
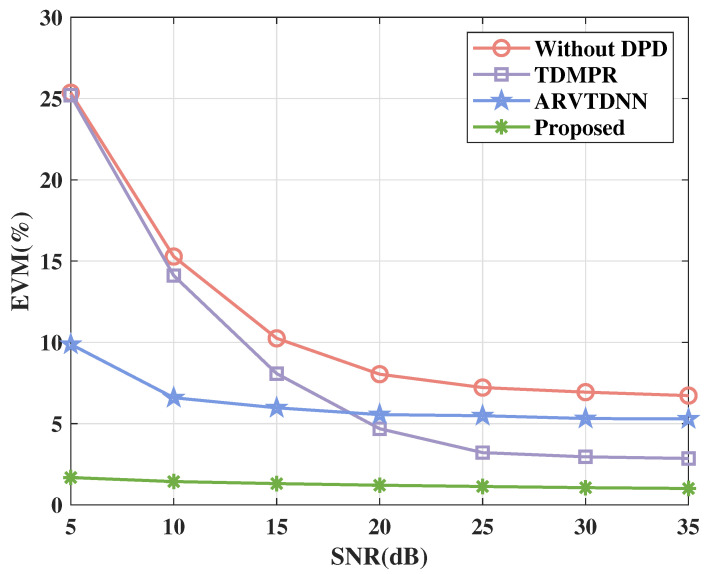
The EVM performance of various DPD methods versus different SNRs.

Figure 7 shows the EVM performance of various DPD methods across different SNRs. It is evident that the scenario without DPD corresponds with deteriorated EVM performance.TDMPR offers only a modest performance improvement compared to the scenario without DPD. The ARVTDNN performs well when the SNR is lower, but it plateaus at around 8% and provides less efficient performance compared to TDMPR when the SNR is above around 18 dB. In contrast, the proposed NN-assisted DPD method delivers outstanding performance across all SNR levels, achieving an EVM of 1.014% at 35 dB.

**Figure 8:**
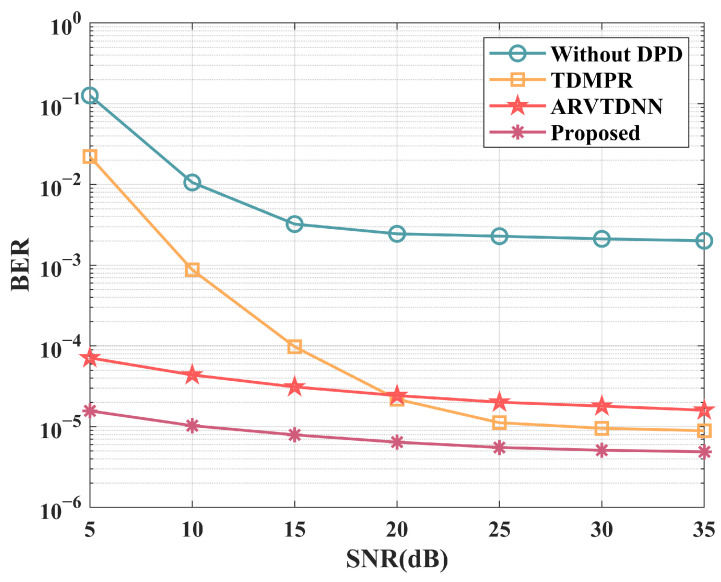
The BER performance of various DPD methods versus different SNRs.

Figure 8 illustrates the bit error rate (BER) of various DPD methods across different SNRs. Specifically, both the proposed method and the ARVTDNN outperform the TDMPR-based method at low SNRs, validating the effectiveness of virtual training in noisy environments. Moreover, the proposed method demonstrates good nonlinearity correction performance at both low and high SNRs. Additionally, the BER performance of the system without DPD saturates easily around $2\times 10^{-2}$, which significantly affects the communication performance of the sub-Nyquist sampling system.

Considering that the nonlinearity of the amplifier is affected by temperature and humidity, three different PA models are simulated in this paper to simulate the nonlinearity of the PA under different temperatures and humidity conditions. We still use the memory polynomial model to model the PA, which is shown in (1). As in [23], from experiments, we drew the conclusion that the higher humidity and temperature generally increase the nonlinear behavior of a PA. Therefore, by modifying the MP coefficients $\left[h_{10},h_{11},h_{12},h_{30},h_{31},h_{32},h_{50},h_{51},h_{52}\right]$, we can simulate various PAs with different levels of nonlinearity: normal nonlinearity for low temperature and low humidity, medium nonlinearity for mild temperature and mild humidity, and severe nonlinearity for high temperature and high humidity. Table 2 demonstrates the model parameters used at different temperatures and humidities.

**Figure 9:**
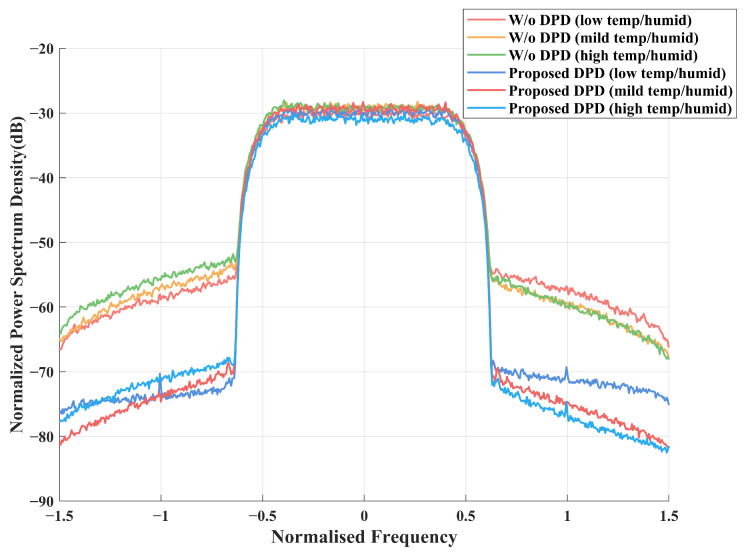
PSD of PA output with the proposed NN-assisted DPD with different levels of temperature and humidity.

**Figure 10:**
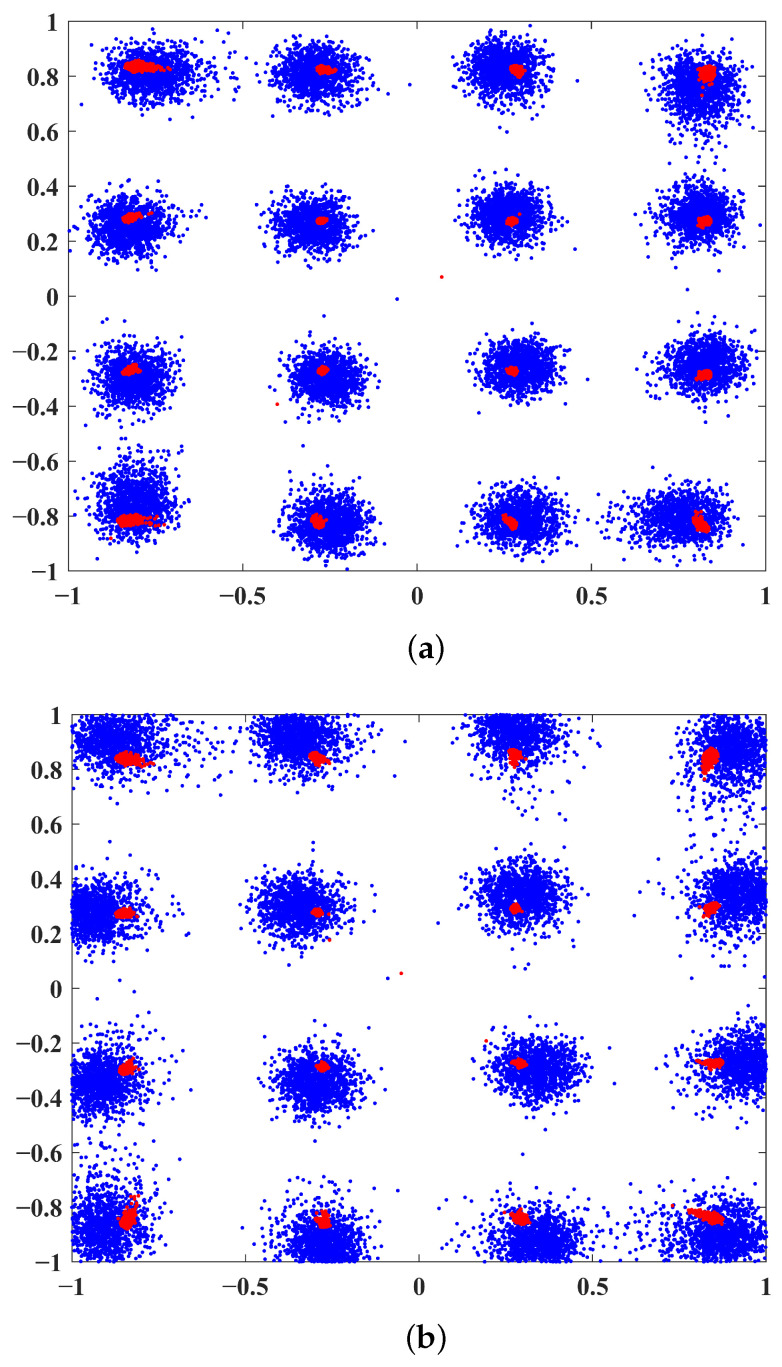

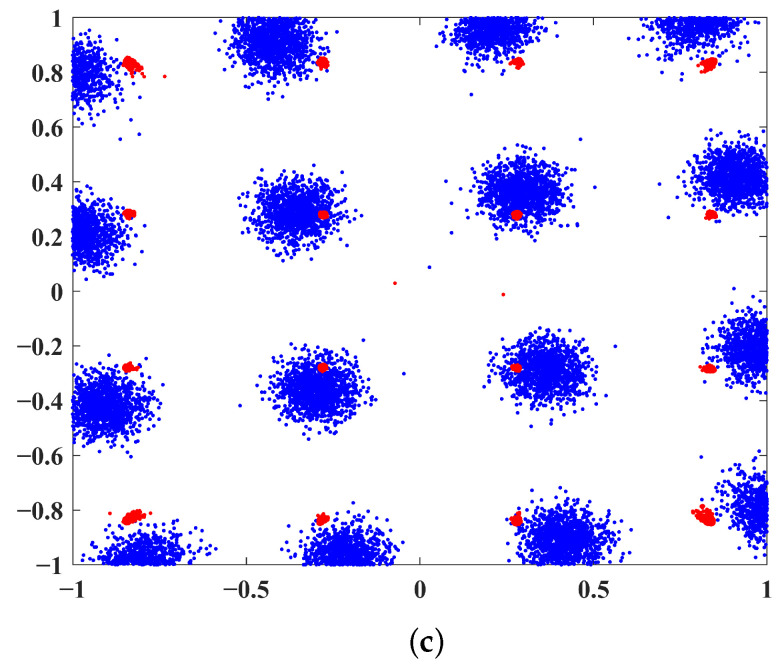
Constellation diagrams at different temperature/humidity levels: (**a**) low temperature/low humidity, (**b**) mild temperature/mild humidity, and (**c**) high temperature/high humidity.

Figure 9 shows the PSD performance of the proposed DPD with three different power amplifiers. It can be seen that the proposed DPD provides excellent performance even with high nonlinearity. In contrast, the performance without considering DPD (denoted as “W/o DPD” in the legends) degrades with the temperature and humidity change. We also simulated the constellation diagrams for these three different power amplifiers under nonlinear conditions. As can be seen in Figure 10a–c, the constellation points are well separated, which demonstrates the robustness and adaptability of the proposed NN-based DPD method.

## 5. Conclusions

In this paper, we proposed an NN-assisted DPD method tailored for sub-Nyquist sampling systems. To address the ambiguity problem resulting from undersampling, a two-stage architecture was introduced. In the first stage, a time delay MP reconstruction-based approach was employed to roughly estimate the broadband DPD nonlinearity using undersampled signals, requiring only a limited number of pilot signals. Subsequently, for the virtual training of DPDs, we proposed an attention-based NN DPD method in the second stage. This learning-based method leverages the transmitted data to learn the reconstructed DPD behaviors, significantly reducing the length of the training sequence and effectively refining the DPD behaviors. Our simulation results demonstrate that the proposed method can effectively mitigate the nonlinear distortion of broadband power amplifiers and provides outstanding performance gain compared to the state-of-the-art methods.

## Figures and Tables

**Table 1:** ACPR and EVM (SNR = 15 dB).

Model	Lower ACPR/dB	Upper ACPR/dB	EVM%
Without DPD	−18.6474	−21.5771	10.25
TDMPR	−22.6645	−22.5484	8.08
Stage 2 only	−34.2174	−34.0762	5.9943
ARVTDNN	−36.3610	−36.0028	5.9746
Proposed	−43.4470	−41.0365	1.3134

**Table 2:** Memory polynomial coefficients for PA modeling.

Simulating Various PA Nonlinearities Due to Temperature Drift and Humidity Variation	Memory Polynomial Coefficients for PA Modeling
Simulate low temperature/low humidity (normal nonlinearity)	[1.0513 + 0.0904j, −0.068−0.0023j, 0.0289 + 0.0054j, −0.0542 − 0.29j, 0.2234 + 0.2317j, −0.0621 − 0.0932j, −0.9657 − 0.7028j, −0.2451 − 0.3735j, 0.1229 + 0.1508j]
Simulate mild temperature/mild humidity (medium nonlinearity)	[1.3883 + 0.1264j, 0.0082 + 0.0090j, −0.0017 − 0.0058j, 0.0996 − 0.2196j, −0.0818 + 0.01696j, −0.0205 + 0.0161j, 0.0071 − 0.0006j, −1.4735 − 1.2327j, −0.0209 − 0.0196j]
Simulate high temperature/high humidity (severe nonlinearity)	[1.9067 + 0.1804j, 0.0075 + 0.0195j, 0.0108 − 0.0024j, −0.1403 + 0.0081j, −0.2134 − 0.0190j, 0.0437 + 0.0081j, −1.0739 − 0.1562j, −0.1196 + 0.1244j, 0.2187 + 0.1474j]

## Data Availability

Data are contained within the article.
